# Does recurrent laryngeal nerve lymph node metastasis really affect the prognosis in node-positive patients with squamous cell carcinoma of the middle thoracic esophagus?

**DOI:** 10.1186/1471-2482-14-43

**Published:** 2014-07-12

**Authors:** Jie Wu, Qi-Xun Chen, Xing-Ming Zhou, Wei-Ming Mao, Mark J Krasna

**Affiliations:** 1 Department of Thoarcic Surgery, Zhejinang Cancer Hospital, 38 Guangji Road, Hangzhou 310022, China; 2 Meridian Cancer Care, Jersey Shore University Medical Center, Neptune, New Jersey, USA

**Keywords:** Esophageal cancer, Lymph node metastasis, Recurrent laryngeal nerve, Squamous cell carcinoma

## Abstract

**Background:**

Recurrent laryngeal nerve (RLN) lymph node metastasis used to be shown a predictor for poor prognosis in esophageal squamous cell carcinoma. The purpose of this study was to evaluate the prognostic impact of RLN node metastasis and the number of metastatic lymph nodes in node-positive patients with squamous cell carcinoma of middle thoracic esophagus.

**Methods:**

A cohort of 235 patients who underwent curative surgery for squamous cell carcinoma of middle thoracic esophagus was investigated. The prognostic impact was evaluated by univariate and multivariate analyses.

**Results:**

Lymph node metastasis was found in 133 patients. Among them, 81 had metastatic RLN nodes, and 52 had at least one positive node but no RLN nodal involvement. The most significant difference in survival was detected between patients with metastatic lymph nodes below and above a cutoff value of six (*P* < 0.001). Multivariate analysis revealed that the number of metastatic lymph nodes was a significant factor associated with overall survival (*P* < 0.001), but RLN lymph node metastasis was not (*P* = 0.865).

**Conclusions:**

RLN Lymph node metastasis is not, but the number of metastatic nodes is a prognostic predictor in node-positive patients with squamous cell carcinoma of the middle thoracic esophagus.

## Background

In esophageal cancer, lymph node metastasis most likely occurs on neck, mediastinum and abdomen. Recurrent laryngeal nerve (RLN) lymph node is located at the cervical base continuous to the upper mediastinum, which is one of the most common sites of lymph node metastasis in thoracic esophageal squamous cell carcinoma [1-4]. The clinical significance of RLN node metastasis in surgical treatment of thoracic esophageal squamous cell carcinoma has been discussed previously. Early metastasis [2,5], initial metastasis [6,7], and even micrometastasis [7] of esophageal squamous cell carcinoma often occur in RLN nodes. In addition, nodal involvement in RLN has been regarded as an indication for three-field lymphadenectomy in the surgical treatment of esophageal cancer [4,8-10]. More importantly, RLN node metastasis has been shown to be a strong predictor of poor prognosis in thoracic esophageal squamous cell carcinoma [3,11].

However, some studies showed that the site of nodal involvement was not associated with the prognosis of thoracic esophageal squamous cell carcinoma, and the number of metastatic lymph nodes had a greater prognostic significance in thoracic esophageal squamous cell carcinoma [12-14]. These results are contradictory to the findings mentioned above that RLN node metastasis is an unfavorable prognostic factor in thoracic esophageal squamous cell carcionoma. To evaluate the outcome of curative esophagectomy treatment, as well as the prognostic impacts of RLN node metastasis and the number of metastatic lymph nodes, in this study, we analyzed a cohort of patients with squamous cell carcinoma of the middle esophagus admitted in our institution.

## Methods

### Patients

Three hundred and twenty six patients with squamous cell carcinoma of the middle thoracic esophagus were surgically treated at the Department of Thoracic Surgery of Zhejiang Cancer Hospital, Hangzhou, China from January 2003 to December 2009. Among these patients, 26 patients with R1 (microscopic residual disease) or R2 (macroscopic residual disease) resections, 48 patients receiving preoperative therapy (chemotherapy and/or radiotherapy), 8 patients with histories of gastric cancer, 5 patients with synchronous cancers (gastric cancer or laryngeal cancer) and 4 patients with non-squamous cell carcinoma of the middle thoracic esophagus were excluded. The records of the remaining 235 patients with curative esophagectomy were retrospectively reviewed. Written informed consents were obtained from all patients before surgery. The Institutional Review Board of Zhejiang Cancer Hospital approved the study and the need for individual patient consent was waived.

The cohort of patients included 194 males and 41 females with an average age of 58 ranging from 37 to 79 years old. Preoperative evaluation included endoscopy with biopsy, barium swallow examination, computerized tomography of the chest and upper abdomen, and ultrasound of the neck. Pulmonary and cardiac function tests were routinely performed to assess medical operability. Histological diagnosis of each of the patients was established before treatment. Tumor location, grade, and stage were defined according to the 7th edition of UICC TNM classification [15]. Recurrent laryngeal nerve palsy and the presence of clinical supraclavicular or cervical nodal involvement were considered a contraindication for surgery.

In our institution, two types of lymphadenectomy were performed for esophageal cancer depending on the operators’ surgical preference. Four surgeons performed 2-field lymphadenectomy, while 2 performed 3-field lymphadenecotmy as a chief operator.

### Surgical procedure

**Figure 1:**
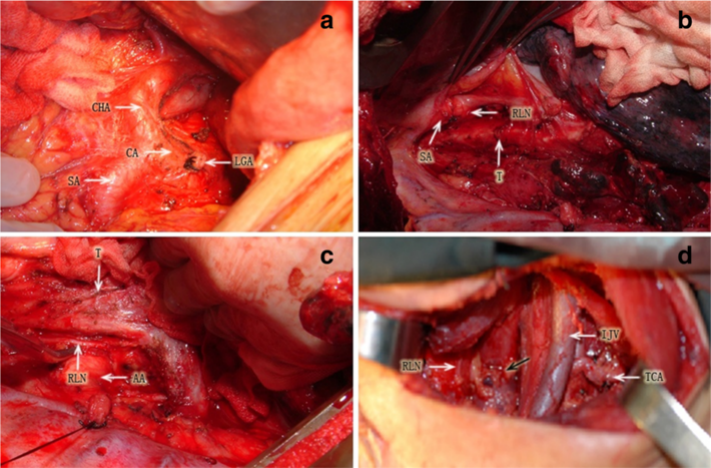
**Lymphadenectomy for esophageal carcinoma. (a)** Upper abdominal field. CA: celiac artery, CHA: common hepatic artery, SA: splenic artery, LGA: left gastric artery. **(b)** Right upper mediastinal field. SA: subclavian artery, RLN: (right) recurrent laryngeal nerve, T: trachea. **(c)** Left upper mediastinal field. RLN: (left) recurrent laryngeal nerve, AA: aotic arch, T: trachea. **(d)** Left cervical field: TCA: transverse cervical artery. IJV: internal jugular vein, RLN: (left) recurrent laryngeal nerve, black arrow: anastomotic site.

A transthoracic esophagectomy was performed for each of the 235 patients with either a 2-field or a 3-field lymphadenectomy. The surgical procedure of esophagectomy with 2-field lymphadenectomy was described previously [16]. In principle, this procedure consisted of esophagectomy with total mediastinal lymphadenectomy through a right thoracotomy, and upper abdominal lymphadenectomy through an upper median laparotomy. Total mediastinal lymphadenctomy was performed according to the classification defined by the International Society for Diseases of the Esophagus (ISDE) [17]. The extent of lymphadenectomy involved dissection of the bilateral RLNs, paratracheal, brachiocephalic artery, paraesophageal, and infraaortic arch nodes, in addition to the middle and lower mediastinal nodes. Upper abdominal lymphadenectomy was performed to include the paracardial, lesser curvature, left gastric, common hepatic, celiac, and splenic nodes. The 3-field lymphadenectomy included cervical lymphadenectomy of the paraesophageal, deep cervical, and supraclavicular nodes in addition to 2-field lymphadenectomy performed through a collar cervical incision. Esophageal anastomosis was performed in the neck for each patient (Figure 1). Gastrointestinal continuity reconstruction was achieved by stomach bypass in 233 patients and by colon conduit in 2 patients. After surgery, the anatomical location of the removed nodes were labeled by the operating surgeon, and then histologically examined with hematoxylin and eosin staining.

### Follow-up

Complete follow-up information was available for all patients. Survival time was defined as the period from the date of surgery till death (including surgical related death and non-cancer related death) or the most recent follow-up in March 2013. The duration of follow-up ranged from 1 month to 131 months (average 45 months, median 37 months). One hundred and sixty four patients died, and the remaining 71 were still alive at the last contact.

### Statistical analysis

Survival curves were constructed using Kaplan-Meier method [18], and log-rank test was used to determine significance [19]. To confirm the optimal cutoff value for the number of metastatic lymph nodes, the Cox proportional hazard model was used to compare survival rates between the groups with fewer and more metastatic lymph nodes [20]. The number of metastatic lymph nodes with the highest χ^2^ value was regarded as the optimal cutoff level. The influence of each clinicopathological variable on survival was assessed using Cox proportional hazard model. A *P* value of less than 0.05 was considered statistically significant.

## Results

### Clinicopatholgoical features

Clinicopathological features of the patients are summarized in Table 1. Of the 235 patients, 159 underwent 2-field and 76 underwent 3-field lymphadenectomy. The majority of patients had T3 disease (157 patients, 67%). Among the 8 patients with T4 tumors, invasions to the lungs were diagnosed in 3 patients, and invasions to the pericardia were diagnosed in 5 patients. A total of 102 patients had no lymph node metastases (43%), and 133 patients had lymph node metastases (57%). Mediastinal and abdominal lymph node metastases were found in 124 (53%) and 46 (20%) patients respectively. Cervical lymph node metastases were found in 23 of 76 (30%) patients who underwent 3-field lymphadenectomy. Of the 133 patients with nodal involvement, 81 (61%) had metastatic RLN nodes and 52 (39%) had at least one positive node but no RLN nodal involvement. The minority of patients (56 patients, 24%) received adjuvant therapy postoperatively.

**Table 1:** Clinicopathological features of the 235 patients with squamous cell carcinoma of the middle thoracic esophagus

**Variables**	**No. (%)**
Age (years)	
< 60	132 (56)
≥ 60	103 (44)
Sex	
Male	194 (83)
Female	41 (17)
Differentiation	
G1	49 (21)
G2	143 (61)
G3	43 (18)
T category	
T1	32 (14)
T2	38 (16)
T3	157 (67)
T4	8 (3)
Node status	
N0	102 (43)
N1	57 (24)
N2	49 (21)
N3	27 (11)
Positive (N+)	133 (57)
RLN -	52 (22)
RLN +	81 (35)
Lymphatic and venous invasion	
No	190 (81)
Yes	45 (19)
Intramural metastasis	
No	220 (94)
Yes	15 (6)
Adjuvant therapy	
No	179 (76)
Yes	56 (24)
Lymphadenectomy type	
2-field	159 (68)
3-field	76 (32)

### The number of metastatic lymph nodes and its stratification

The number of metastatic lymph nodes of the 133 patients ranged from 1 to 32, with a mean of 4.4 and a median of 3. The Cox proportional hazards regression model revealed that the most significant difference in survival was identified with a cutoff value of six metastatic lymph nodes, yielding a χ^2^ value of 20.903, a hazard ratio of 2.820, and a 95% confidence interval of 1.774-4.482 (Table 2).

**Table 2:** Cutoff values for the number of metastatic lymph nodes analyzed by Cox proportional hazard model

**Cut-off values**	**χ**^**2**^	**Hazards ratio (95% CI)**	***P *** **value**
1 vs. ≥2	2.758	1.457 (0.932-2.278)	0.099
2 ≤ vs. ≥3	5.706	1.599 (1.084-2.359)	0.018
3 ≤ vs. ≥4	4.042	1.486 (1.008-2.191)	0.046
4 ≤ vs. ≥5	8.854	1.804 (1.209-2.692)	0.004
5 ≤ vs. ≥6	19.610	2.542 (1.658-3.898)	<0.001
6 ≤ vs. ≥7	20.903	2.820 (1.774-4.482)	<0.001
7 ≤ vs. ≥8	15.544	2.269 (1.597-4.330)	<0.001
8 ≤ vs. ≥9	6.543	2.070 (1.171-3.660)	0.012
9 ≤ vs. ≥10	6.696	2.189 (1.191-4.023)	0.012
10 ≤ vs. ≥11	2.698	1.766 (0.888-3.514)	0.105

### Survival

**Figure 2:**
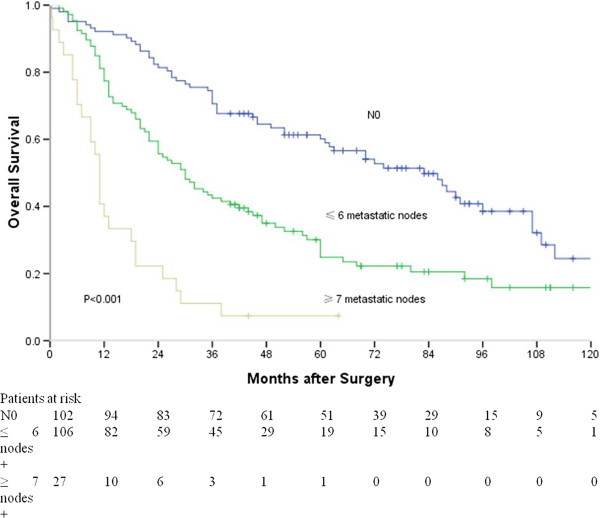
**Survival curves of patients with various number of metastatic nodes (without lymph node metastasis vs. with ≤ 6 metastatic nodes, *P*** **< 0.001; with ≤ 6 metastatic nodes vs. with ≥ 7 metastatic nodes, *P*** **< 0.001; without lymph node metastasis vs. with ≥ 7 metastatic nodes, *P*** **< 0.001).**

The median survival for all patients was 37 months, and the 1-, 3- and 5-year survival rates were 79%, 51%, and 39%, respectively. The Kaplan-Meier curves constructed using the optimal values for the number of metastatic lymph nodes are shown in Figure 2. The median survival time of patients without lymph node metastasis, with ≤ 6 metastatic lymph nodes, and with ≥ 7 metastatic lymph nodes were 83, 30 and 11 months, respectively. There were significant differences between patients without lymph node metastasis and with ≤ 6 metastatic lymph nodes (*P* < 0.001), between patients without lymph node metastasis and with ≥ 7 metastatic lymph nodes (*P* < 0.001), and between patients with ≤ 6 metastatic lymph nodes and with ≥ 7 metastatic lymph nodes (*P* < 0.001).

**Figure 3:**
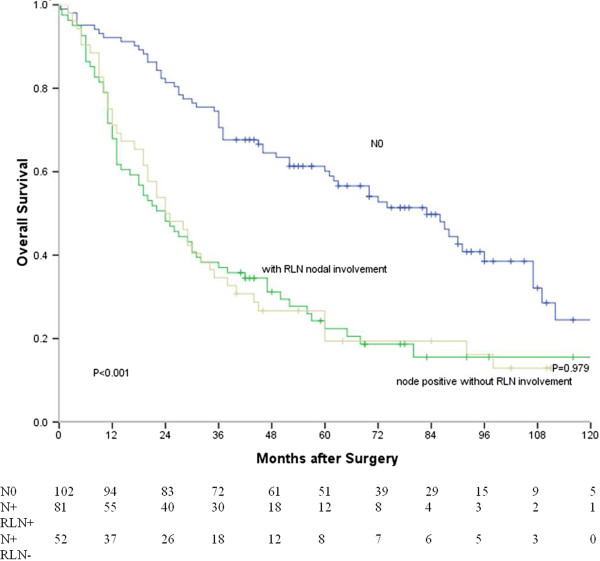
**Survival curves of patients with different RLN node status (without lymph node metastasis vs. with RLN nodal involvement, *P*** **< 0.001; without lymph node metastasis vs. node-positive without RLN nodal involvement, *P*** **< 0.001; with RLN nodal involvement vs. node-positive without RLN nodal involvement, *P*** **= 0.979).**

Survival curves based on lymph node status are shown in Figure 3. The median survival time of node-negative patients, node-positive patients without RLN nodal involvement and RLN node-positive patients were 83, 24 and 24 months, respectively. There were significant differences between node-negative patients and RLN node-positive patients (*P* < 0.001), and between node-negative patients and node-positive patients without RLN nodal involvement (*P* < 0.001). There was no significant difference between RLN node-positive patients and node-positive patients without RLN nodal involvement (*P* = 0.979).

**Figure 4:**
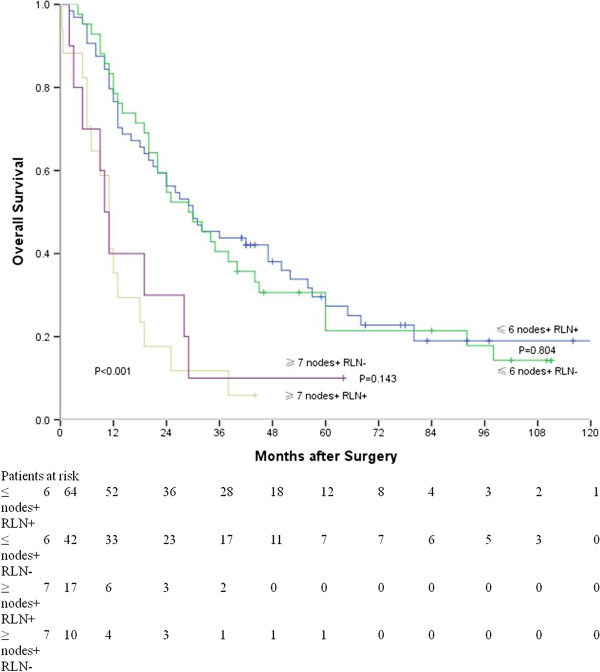
**Survival curves of patients with various number of metastatic nodes and different recurrent laryngeal nerve node status (≤ 6 nodes + RLN + vs. ≤ 6 nodes + RLN-, *P*** **= 0.928; ≥ 7 nodes + RLN- vs. ≥ 7 nodes + RLN+, *P*** **= 0.520).**

Furthermore, the difference in survival time of patients with ≤ 6 metastatic lymph nodes was insignificant between RLN node-positive patients and node-positive patients without RLN nodal involvement. Similarly, the difference in survival between the two groups mentioned above and patients with ≥ 7 metastatic lymph nodes was also insignificant (*P* = 0.804) (*P* = 0.143) (Figure 4).

**Figure 5:**
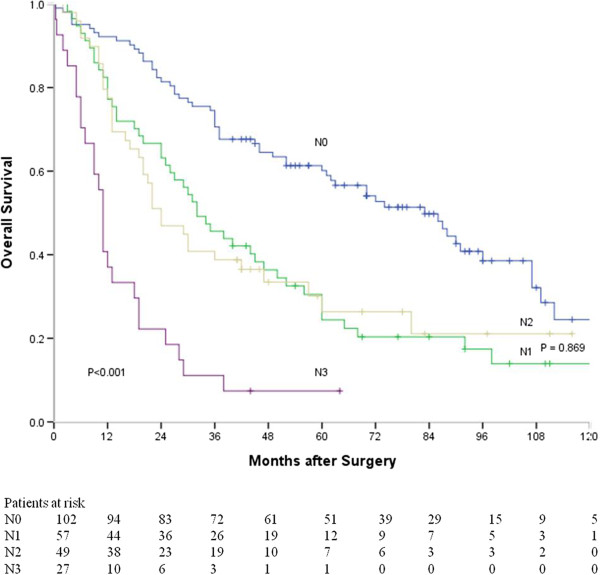
**Survival curves of patients with different N stages (N0 vs. N1, *P*** **< 0.001; N0 vs. N2, *P*** **< 0.001; N0 vs. N3, *P*** **< 0.001; N1 vs. N2, *P*** **= 0.869; N1 vs. N3, *P*** **< 0.001; N2 vs. N3, *P*** **< 0.001).**

In addition, survival curves based on N stages according to the 7th edition of UICC TNM classification are shown in Figure 5. The median survival time of N0, N1, N2, and N3 patients were 83, 32, 24, and 11 months respectively. There was a significant difference in survival time among all these patients (*P* < 0.001). However, the difference in survival time was insignificant between N1 and N2 patients (*P* = 0.869).

### Univariate and multivariate analyses for clinicopathological variables

In a univariate analysis for survival, T category (*P* < 0.001), node status (*P* < 0.001), lymphatic and venous invasion (*P* = 0.001), intramural metastasis (*P* = 0.009) and adjuvant therapy (*P* = 0.034) were significantly associated with overall survival (Table 3). Because three methods (N stage, number of metastatic nodes, RLN node metastasis) were used in stratifying N status, three Cox models were constructed to avoid problems with the presence of multicollinearity. As shown in Table 4, the number of positive nodes (*P* < 0.001) were identified as a significant factor associated with overall survival, while RLN node metastasis was not a prognostic predicator (*P* = 0.865). In a model with stratification by N stage, N2 stage was insignificantly associated with overall survival (*P* = 0.722).

**Table 3:** Univariate analysis of 235 patients with squamous cell carcinoma of the middle thoracic esophagus

**Variables**	**No. of patients**	**Survival (%) 1y 3y 5y**	**Median survival time (months)**	***P *** **value**
Age (years)				0.771
< 60	132	78 52 40	37	
≥ 60	103	81 51 37	42	
Sex				0.206
Male	194	79 50 38	36	
Female	41	71 59 43	60	
Differentiation				0.080
G1	49	88 55 49	45	
G2	143	81 53 40	42	
G3	43	65 40 36	24	
T category				<0.001*
T1/T2	70	91 71 62	86	
T3/T4	165	74 42 29	29	
Node status				<0.001*
N0	102	92 71 60	83	
N1	57	77 46 24	32	
N2	49	78 39 26	24	
N3	27	37 11 7	11	
Node status				<0.001*
N0	102	92 71 60	83	
N + RLN-	52	71 35 19	24	
N + RLN+	81	68 37 22	24	
Node status				<0.001*
N0	102	92 71 60	83	
≤ 6 positive nodes (N+)	106	77 43 25	30	
≥ 7 positive nodes (N+)	27	37 11 7	11	
Lymphatic and venous invasion				<0.001*
No	190	82 56 42	44	
Yes	45	67 31 23	22	
Intramural metastasis				0.009*
No	220	81 53 40	42	
Yes	15	60 20 20	18	
Adjuvant therapy				0.034*
No	179	79 53 44	46	
Yes	56	79 45 19	25	
Lymphadenectomy type				0.271
2-field	159	80 54 41	45	
3-field	76	78 45 35	33	

*Variables were also used for multivariate analysis.

**Table 4:** Multivariate analysis for 133 node-positive patients with squamous cell carcinoma of the middle thoracic esophagus

**Variables**	**Hazard ratio**	**95% CI**	***P *** **value**
Model 1			
N1	1.000 (reference)		
N2	1.084	0.696-1.688	0.722
N3	3.135	1.877-5.236	<0.001
Model 2			
N + RLN-	1.000		0.865
N + RLN+	1.035	0.693-1.548	
Model 3			
≤ 6 positive nodes (N+)	1.000 (reference)		<0.001
≥ 7 positive nodes (N+)	3.022	1.888-4.837	

### RLN node status and the type of lymphadenectomy

In the 81 patients with RLN node metastasis, the difference in survival rate was insignificant between 2-field and 3-field lymphadenectomy (*P* = 0.843). In the other 154 patients without RLN node metastasis, survival rate did not differ significantly between 2-field and 3-field lymphadenectomy (*P* = 0.661).

## Discussion

Here we demonstrated that the presence of RLN node metastasis was not a prognostic predicator in node-positive patients with squamous cell carcinoma of the middle thoracic esophagus. A previous report including 55 patients with esophageal squamous cell carcinoma who underwent esophagectomy with 2-field lymphdenectomy showed that RLN node metastasis was the strongest prognostic predicator [11]. That report was more heterogeneous in term of tumor site: tumors were located below and above the carina in 40 and 15 patients, respectively [11]. Different tumor sites might lead to different frequencies of lymph node metastasis. In that report, frequencies of RLN node metastasis was 18% in all patients (10/55) and 26% (10/34) in node-positive patients [11]. While in our study, the frequencies of RLN node metastasis were 34% (81/235) in all patients and 62% (81/133) in node-positive patients. More importantly, Authors only performed univariate analysis, but did not perform multivariate analysis in that cohort [11]. Dealing with data this way may cause confounding effect that influenced the interpretation of results. There was another report on clinical outcomes of 106 patients with esophageal squamous cell carcinoma who underwent 3-field lymphadenectomy [3]. Univariate and multivariate analyses indicated that RLN node metastasis was the most unfavorable prognostic factor [3]. In that series, 10, 67 and 29 patients had tumors located in the upper, middle and lower thoracic esophagus, respectively. Although RLN node metastasis occurred in 60 of 78 (77%) node-positive patients, the report did not state how many patients with lesions in the middle thoracic esophagus had RLN node metastasis. Furthermore, the factor of the number of metastatic lymph nodes was not included in the analysis [3].

Among various possible prognostic predicator of esophageal carcinoma, the importance of number of metastatic nodes has been widely recognized [1,10,11,13,21]. Patients with a large number of metastatic nodes had a lower average survival rate than those with less metastatic nodes. Stratification of the number of metastatic nodes varied in different reports (for example, 1–3 vs ≥ 4 [11,13], 1–4 vs ≥ 5 [10], 1–5 vs ≥ 6 [21], and 1–7 vs ≥ 8 [1]). Our report showed that the survival rate decreased with an increasing number of metastatic nodes, and that the optimal cutoff value was between 1–6 and ≥ 7 metastatic nodes. On the other hand, there was little evidence supporting that the site of metastatic nodes influenced the prognosis of esophageal carcinoma [14,22,23]. For example, celiac node metastasis, which was regarded as M1 disease in the past, did not mean poor prognosis in node-positive patients with esophageal cancer [22,23]. It was found that for middle and lower thoracic esophageal carcinoma, survival of patients with celiac node metastasis did not differ from those with left gastric node metastasis [23]. The 7th edition of TNM staging system also has redefined a regional node of esophageal cancer as any periesophageal lymph nodes from cervical nodes to celiac nodes; yet N staging has already been subclassfied according to the number of metastatic nodes [15].

The frequency of RLN node metastasis was reported between 20% and 50% in patients with squamous cell carcinoma of the upper and middle thoracic esophagus [1,2,4,8]. In our institution, upper thoracic tumor is routinely treated with radiotherapy-dominated multidisciplinary therapy. Some authors pointed out that RLN was the initial metastatic site (including micrometastatic site) in esophageal squamous cell carcinoma [6,7]. Others found that the histology of RLN node was characterized by large cortical area without anthracosis and hyalinization, which suggests a high filtration activity [5]. All these features of RLN nodes need to be further investigated. Some authors found that the prognoses of patients with RLN node metastasis was better in the three-field lymphadenectomy group than in the two-field lymphadenectomy group, while in patients without RLN node metastasis, there was no significant differences in survival between these two groups [8]. Their results could not be duplicated in this study. It should be noted that the features of patients in that study including age, tumor location and disease stage, differed between patients with RLN node metastasis and those without RLN node metastasis [8]. These differences between patients groups could cause biased results. Frequency of cervical nodal metastasis (30%) in this report was similar to previous reported. Significant associations between RLN node metastasis and cervical node metastasis in esophageal squamous cell carcinoma were emphasized by many authors, and they firmly believed that 3-field lymphadenectomy was indicated if RLN node metastasis happens [4,8-10]. But there is lack of high-level evidence supporting 3-field lymphadenectomy in terms of long-term survival [13,24,25]. Instead it is certain that increased postoperative morbidity and impaired long-term quality of life are associated with 3-field lymphdenectomy [24,25]. Although 3-field lymphadenectomy might offer survival benefit for selected patients with esophageal cancer, the controversy over the optimal extent of lymphadenectomy still exists [25,26]. For a majority of patients there would be no arguments about performing two-field lymphadenectomy to offer a balance between benefits and risks. In addition, the emphasis of three-field lymphadenectomy lies more in RLN lymphadenectomy than in cervical lymphadenectomy [24]. In this study, 3-filed lymphadenetomy did not show its survival benefits compared with 2-field lymphadenectomy, but RLN node metastasis also did not portend a worse prognosis in node-positive patients. Thus lymphadenectomy including dissection of RLN nodes is strongly supported.

Several potential shortcomings of the present study are worth mentioning. This retrospective study from a single institution suffers from the typical biases associated with such studies. The choice of surgical procedures depended on surgeons’ preference without strict criteria. It is likewise unavoidable that lymphadenectomy was performed in more or less different extent by different surgeons. In addition, there was no set standard for patients to receive adjuvant therapy. As shown in the result of the univariate analysis, patients with adjuvant therapy had worse survival than those without adjuvant therapy. The majority of patients with adjuvant therapy had a large number of metastatic nodes (data not shown). However, this series was proved to be homogenous in clinical variables including tumor site and pathologic type. Further multi-institutional studies with larger sample size are needed to confirm these results.

## Conclusions

RLN lymph node metastasis is not a prognostic predictor in node-positive patients with squamous cell carcinoma of the middle thoracic esophagus. However, the number of metastatic nodes is a key prognostic predictor. Systemic lymphadenectomy including dissection of RLN nodes is therefore necessary for these patients.

## Competing interests

The authors have no conflicts of interest to disclose.

## Authors’ contributions

JW conceived this study, collected data, performed analysis and drafted the manuscript. QXC participated in study design, literature search and coordination. JW, QXC, XMZ and WMM participated in the treatment of these patients. MJK performed data analysis and helped to draft the manuscript. All authors read and approve the final manuscript.

## Pre-publication history

The pre-publication history for this paper can be accessed here:

http://www.biomedcentral.com/1471-2482/14/43/prepub
